# Magnetic Nanoclusters Increase the Sensitivity of Lateral Flow Immunoassays for Protein Detection: Application to Pneumolysin as a Biomarker for *Streptococcus pneumoniae*

**DOI:** 10.3390/nano12122044

**Published:** 2022-06-14

**Authors:** María Salvador, José Luis Marqués-Fernández, Alexander Bunge, José Carlos Martínez-García, Rodica Turcu, Davide Peddis, María del Mar García-Suárez, María Dolores Cima-Cabal, Montserrat Rivas

**Affiliations:** 1 Department of Physics & IUTA, University of Oviedo, Campus de Viesques, 33203 Gijón, Spain; salvadormaria@uniovi.es (M.S.); uo254204@uniovi.es (J.L.M.-F.); jcmg@uniovi.es (J.C.M.-G.); 2 Institute of Structure of Matter—National Research Council (CNR), 00016 Monterotondo Scalo (RM), Rome, Italy; davide.peddis@unige.it; 3 National Institute for Research and Development of Isotopic and Molecular Technologies, 400293 Cluj-Napoca, Romania; alexander.bunge@itim-cj.ro (A.B.); rodica.turcu@itim-cj.ro (R.T.); 4 Department of Chemistry and Industrial Chemistry, Università degli Studi di Genova, 16146 Genova, Italy; 5 Escuela Superior de Ingeniería y Tecnología (ESIT), Universidad Internacional de la Rioja (UNIR), Avenida de la Paz, 137, 26006 Logroño, Spain; mar.garcia.suarez@unir.net (M.d.M.G.-S.); dolores.cima@unir.net (M.D.C.-C.)

**Keywords:** lateral flow immunoassays, pneumococcal pneumonia, COVID-19, magnetic nanoparticles, magnetic separation, inductive sensor

## Abstract

Lateral flow immunoassays for detecting biomarkers in body fluids are simple, quick, inexpensive point-of-care tests widely used in disease surveillance, such as during the coronavirus disease 2019 (COVID-19) pandemic. Improvements in sensitivity would increase their utility in healthcare, food safety, and environmental control. Recently, biofunctional magnetic nanoclusters have been used to selectively label target proteins, which allows their detection and quantification with a magneto-inductive sensor. This type of detector is easily integrated with the lateral flow immunoassay format. Pneumolysin is a cholesterol-dependent cytolysin and one of the most important protein virulence factors of pneumonia produced by *Streptococcus pneumoniae*. It is recognized as an important biomarker for diagnosis in urine samples. Pneumonia is the infectious disease that causes the most deaths globally, especially among children under five years and adults over 65 years, most of them in low- and middle-income countries. There especially, a rapid diagnostic urine test for pneumococcal pneumonia with high sensitivity and specificity would be helpful in primary care. In this work, a lateral flow immunoassay with magnetic nanoclusters conjugated to anti-pneumolysin antibodies was combined with two strategies to increase the technique’s performance. First, magnetic concentration of the protein before the immunoassay was followed by quantification by means of a mobile telephone camera, and the inductive sensor resulted in detection limits as low as 0.57 ng (telephone camera) and 0.24 ng (inductive sensor) of pneumolysin per milliliter. Second, magnetic relocation of the particles within the test strip after the immunoassay was completed increased the detected signal by 20%. Such results obtained with portable devices are promising when compared to non-portable conventional pneumolysin detection techniques such as enzyme-linked immunosorbent assays. The combination and optimization of these approaches would have excellent application in point-of-care biodetection to reduce antibiotic misuse, hospitalizations, and deaths from community-acquired pneumonia.

## 1. Introduction

Pneumonia is the leading cause of death from infectious diseases globally [1]. Moreover, its high incidence places it, along with other lower respiratory diseases, as the third overall global cause of mortality, surpassed only by ischemic heart and cerebrovascular diseases [2]. Pneumonia incidence is most significant at the extremes of age. People at risk are the elderly (>65 years old), especially those with comorbidities, and children (<5 years old), for whom it is the leading cause of mortality after the neonatal period. This is especially concerning because most pneumonia deaths should be preventable [3]. Even when it is not fatal, it increases the risk of death for an extended period after its onset [4]. A growing aging population and an increase in comorbidities [5] make this prospect even worse, with considerable implications for healthcare systems worldwide [6].

Identification of the etiologic agent of pneumonia is essential to guide therapy (avoiding antibiotics over-prescription) and prevention and control strategies; bacteria, viruses, and fungi can all cause pneumonia [7]. *Streptococcus pneumoniae* (pneumococcus) remains the leading pathogen responsible for most community-acquired pneumonia and other respiratory and systemic infections such as meningitis [8]. Pneumonia is currently the most common co-infection in COVID-19 patients, which likely contributes significantly to morbidity and mortality [9,10].

Usually, the diagnosis of pneumococcal pneumonia is based on Gram stain and sputum or blood cultures. Unfortunately, appropriate samples of the former are difficult to obtain, especially from pediatric patients, and the latter has a surprisingly poor yield [11]. Polymerase chain reaction (PCR) technology can be applied to detect the bacteria [12], with limits of detection as low as 10^3^ genome copies per mL [13]. However, it requires sophisticated equipment and DNA kits that are likely unaffordable for primary healthcare centers and poorly resourced locations worldwide. Another option is the commercial lateral flow test Binax Now^®^ for the detection of pneumococcal polysaccharide C in urine. However, it has been reported to have low sensitivity in adults, probably associated with the serotype changes and their polysaccharide C variability [14], and it is not helpful in children if they have had a prior bronchopulmonary disease or if they have been vaccinated [15,16]. Therefore, the current challenge in pneumonia diagnosis, mainly but not exclusively in children, is a rapid diagnostic test with high sensitivity and specificity in handy samples such as urine. Alone or in combination with others, it would enable precise diagnosis and prompt treatment, a reduction in hospitalization, avoidance of empirical antibiotic prescriptions and increasing antibiotic resistance, and, especially in less-developed countries, prevention of fatal outcomes.

Pneumolysin (PLY) is a 53 kDa protein and one of the most studied virulence factors of pneumococcus, the most important at the protein level. PLY is a protein toxin belonging to the family of thiol-activated toxins, also called cholesterol-dependent cytolysins. It is produced by more than 20 species of Gram-positive bacteria, which are characterized by forming pores in the membranes of eukaryotic cells through their binding to cholesterol, causing cell destruction [17,18]. The PLY conserved sequence is expressed in almost all pneumococcal isolates. Its role in pneumococcal pathogenicity has been extensively studied; mutant bacteria lacking PLY expression prove its relevant function in the infection [19,20]. This has led several authors to study its potential role as a therapeutic target of pneumococcal infection through the use of molecules that directly or indirectly block its mechanism of action [21]. The role of PLY as a diagnostic target is less explored. Our previous studies have demonstrated its importance as a potential biological marker of pneumococcal infection since its detection is a symptom of the disease [22]. Hence, different enzyme-linked immunosorbent assay (ELISA) tests for PLY detection in the urine of sick patients have been developed [23,24], with the lowest limit of detection being 2.3 pg/mL for the chemiluminescent assay. However, these tests require particular and expensive reagents and equipment, making establishing them in poorly resourced locations difficult.

Lateral flow immunoassays (LFAs) are bio-testing methods with many advantages for point-of-care (POC) applications, such as minimal manipulation, quickness, portability, easy use, and low cost. Their introduction to the general market occurred in the early 1980s with the home pregnancy test [25], and the COVID-19 pandemic has made them more familiar. Besides these applications, they have been successfully used to detect other targets in different types of specimens [26,27].

LFAs depend on capillary action and biorecognition. A strip of nitrocellulose guides the liquid sample by capillarity, and the target molecule is selectively trapped at a test line by an immunological reaction. The biomolecule is labeled with nanoparticles to make it detectable. These nanoparticles (gold or latex) give a color signal that provides a yes/no (presence/absence) response. Such a qualitative result is sufficient for many applications; however, many others need quantitative results for cancer diagnosis and prognosis, serological information, or comparison against toxin thresholds. The quantification of the analyte can be accomplished by optical transducers based on image analysis or reflectance. To our knowledge, only one LFA employing plasmonic Surface-Enhanced Resonance Raman Scattering (SERSS) has been published [28], with a limit of detection of 3.6 pg/mL. However, the signal quantification system by SERSS is difficult to implement for portable and decentralized detection devices. Furthermore, optical methods are sensitive to interference from the ambient light, humidity, and staining or aging of the paper strip, which frequently cause difficulties in calibration and reproducibility [27,29]. Additionally, only nanoparticles at the membrane surface contribute significantly to the signal [30].

Magnetic nanoparticles have been used before as efficient labels in LFAs [31]. They can be easily tailored in terms of size, surface, and magnetic properties. Magnetic sensors can detect them without interference from the biological sample or the paper. More interestingly, these magnetic properties can be used for external manipulation, pre-concentration, or separation of the target analyte from the sample matrix. These strategies can improve the sensitivity of the LFAs enormously [32,33].

Our research group has developed a detection method using superparamagnetic nanoparticles and an inductive sensor, which does not compromise the advantages of the LFAs. It is based on an excitation detection planar coil that avoids the need for additional excitation coils or bulky components [34,35]. It is an affordable system that can be easily miniaturized in a POC device. Previous studies have shown that the magnetic labels’ crucial properties are their initial magnetic susceptibility and the mass retained in the test line [36]. From this point of view, clusters of magnetic nanoparticles are advantageous over isolated particles.

For this work, we developed an LFA to detect PLY with biofunctional magnetic nanoclusters (MNCs) conjugated to an anti-PLY antibody. The polyol solvothermal synthesis method was optimized for reproducible and scalable production, yielding MNCs with large initial magnetic susceptibility and high magnetic moment. A considerable improvement in the LFA’s sensitivity was achieved thanks to the multifunctionality of the MNCs. First, magnetic preconcentration and relocation decrease the detection limit. Second, MNCs enable dual detection: optical (the test line has a brownish color) and magnetic (detectable, for example, with an inductive sensor).

## 2. Materials and Methods

### 2.1. Chemicals and Reagents

Sodium polyacrylate (molecular weight ≈ 2100), FeCl_3_·6H_2_O, anti-mouse IgG (M8642-1MG), 1-ethyl-3-[3-di-methylaminopropyl] carbodiimide (EDC), N-hydroxysuccinimide (NHS), bovine serum albumin (BSA), 2-(N-morpholino) ethanesulfonic acid (MES), and Tween20 were purchased from Sigma-Aldrich (Madrid, Spain). Neutravidin protein was obtained from Thermo Fischer Scientific (Waltham, MA, USA). Diethylene glycol 99% and ethylene glycol 99% were obtained from Alfa Aesar (Kandel, Germany) and S.C. Simex SRL (Gherla, Romania), respectively. Ethanol pure was acquired from Chemical Company S.A. (Iași, Romania) First, 10 mM phosphate-buffered saline (PBS) containing NaH_2_PO_4_ (0.065 g) and Na_2_HPO_4_ (0.2875 g) was prepared using ultrapure water, and its pH was adjusted to 7.4 by the addition of HCl (1 M) or NaOH (1 M). Further dilutions were prepared using ultrapure water.

Recombinant pneumolysin protein was produced following the procedure described in [37]. The *ply* gene was cloned into the pTrc 99A plasmid and the protein expressed in *Escherichia coli JM105*. The toxin was purified from the cellular lysates by using a HiLoad 16/10 phenyl-Sepharose hydrophobic column. Anti-pneumolysin monoclonal antibodies (PLY-4 used as a capture antibody and PLY-7 as a detection antibody) were provided by Scientific-Technical Services of the University of Oviedo and produced as previously described in [38]. Briefly, BALB/c mice were subjected to successive rounds of immunization with 10 mg of the toxin in 200 mL of complete Freund’s adjuvant. Splenocytes from immunized animals were fused to Sp2/0 cells at a ratio of 1:1 by standard methods, and selected hybridomas were subcloned twice. IgGs from the supernatants were purified by affinity chromatography on protein A fast flow columns, and their purity and reactivity were assessed by sodium dodecyl sulphate–polyacrylamide gel electrophoresis and enzyme-linked immunosorbent assay.

For the lateral flow strips, the backing cards (HF000MC100) and the glass fiber membrane (GFCP001000) used as a sample pad were purchased from Merck Life Science (Madrid, Spain). The nitrocellulose membranes (UniSart CN95) were provided by Sartorius, Madrid, Spain, and the absorbent pads (CF5) by Cytiva Europe GmbH, (Madrid, Spain). All these components were used without further modification (neither washed nor blocked).

### 2.2. Magnetic Nanoclusters Synthesis and Characterization

The MNC synthesis was performed by a solvothermal polyol process similar to [39], but with several changes. A mixture of ethylene glycol (20 mL) and diethylene glycol (40 mL) was used to suspend/dissolve sodium acetate (6 g) and sodium polyacrylate (6 g). FeCl_3_·6H_2_O (2.17 g) was dissolved in diethylene glycol (20 mL). After stirring for 10 min at room temperature, the iron chloride solution was added to the suspension/solution of acetate and polyacrylate, stirred well for another 10 min, and transferred into a Teflon-lined autoclave (200 mL capacity). The autoclave was heated to 200 °C for 15.5 h and cooled naturally. The magnetite clusters were rinsed into a beaker with ethanol, magnetically separated, and washed two times with ethanol and three times with water before their re-suspension in water. The concentration of magnetic clusters was 22 mg/g suspension.

The size and shape of the nanoclusters were examined by scanning transmission electron microscopy (STEM) and high-resolution transmission electron microscopy (HRTEM). STEM images were acquired with a Hitachi HD2700 (Hitachi Ltd., Tokyo, Japan) equipped with a cold field emission gun and a dual energy-dispersive X-ray system (X-Max N100TLE silicon drift detector) from Oxford Instruments. For the analysis, a suspension of the samples was previously sonicated (<10 s) with a UP100H ultrasound finger and deposited by the droplet method on a 400-mesh copper grid coated with a thin carbon layer. The nominal operating voltage was 200 kV. HRTEM images were obtained with a JEOL JEM-2100 200 kV (JEOL USA Inc., Peabody, MA, USA).

The analysis of the surface chemical composition of the clusters was performed by Fourier transform infrared (FT-IR) and X-ray photoelectron (XPS) spectroscopy. FT-IR spectra were recorded using a JASCO FTIR 4600A spectrophotometer with ATR-PRO-ONE accessory (JASCO Deutschland GmbH, Pfungstadt, Germany). The spectrum was CO_2_-, H_2_O-, ATR-, and baseline-corrected. XPS spectra were obtained using a SPECS XPS spectrometer (SPECS Surface Nano Analysis GmbH, Berlin, Germany) equipped with a dual-anode Al/Mg X-ray source, a PHOIBOS 150 2D CCD hemispherical energy analyzer, and a multi-channel detector with vacuum maintained at 1.3 × 10^−10^ kPa. The Al Kα X-ray source (1486.6 eV) operated at 200 W was used for the XPS investigations. XPS survey spectra were recorded at 30 eV pass energy, 0.5 eV/step. The high-resolution spectra for individual elements were recorded by accumulating multiple scans at 30 eV pass energy and 0.1 eV per step.

DC magnetization measurements were performed using a vibrating sample magnetometer (Cryogenic Ltd., London, UK). Zero field cooling (ZFC) and field cooling (FC) curves were obtained using a Quantum Design SQUID magnetometer (Quantum Design, San Diego, CA, USA). Zero-field-cooled ($M_{\mathrm{ZFC}}$) and field-cooled ($M_{\mathrm{FC}}$) magnetization measurements were carried out by cooling the sample from room temperature to 5 K in zero magnetic field; then, a static magnetic field of 2.5 mT was applied. $M_{\mathrm{ZFC}}$ was measured during warming from 5 to 300 K, whereas $M_{\mathrm{FC}}$ was recorded during the subsequent cooling. The magnetization values were referenced to the mass of iron oxide measured by thermogravimetric analysis.

**Figure 1:**
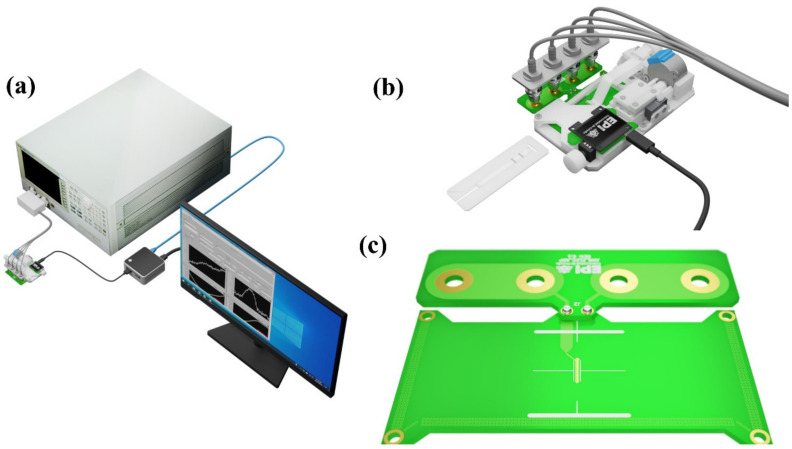
(**a**) Schematic representation of the sensing system showing the impedance analyzer, the micropositioner, and the personal computer used to control both. (**b**) Image of the sensing device, which comprises the planar inductor, the LFAs holder containing an LFA sample, and the micropositioner. The image is represented to scale. (**c**) Detailed image of the planar inductor specifically designed to encompass the entire width of the LFAs during scanning.

The assessment of the MNCs as reporters in the inductive sensor was performed by depositing some droplets of known mass onto a 10 mm × 2 mm piece of blotting paper and left to dry for at least 12 h. The homemade inductive sensor has been specially adapted for scanning the samples (see Figure 1). A detailed description of the sensor can be found elsewhere [40,41]. It consists of a planar inductor whose impedance magnitude and phase are continuously measured with a precision impedance analyzer (Agilent 4294A, 16048G test leads, 500 mV/20 MHz, Keysight Technologies, Santa Rosa, CA, USA) while the sample is micropositioned and displaced in steps of 0.1 mm. The planar coil is constructed as a printed circuit board (PCB), which was produced by a commercially available industrial process. The substrate of the PCB is FR-4 Standard Tg 130–140/Tg 155 of 1.6 mm. The tracks in the sensitive area are made of a 0.018 mm thick layer of copper, deposited by an electroless process, and etched by cupric chloride. The surrounding areas of the tracks are filled with a photoactivated epoxy that flattens the surface. The specific dimensions of the planar coil are a two-turn planar inductor with a track width of 0.15 mm and a 0.15 mm track separation. The sensitive area, composed exclusively of parallel tracks, has dimensions of 1.4 mm × 12 mm. These dimensions cover the sample in the vertical direction but not along the longitudinal one. This longitudinal dimension is covered by the sample scanning.

The positioning system keeps the sample and the sensor surface in close contact during the scan. The pressure between both is 0.9 N, neglecting the sample weight. The high magnetic permeability of the particles induces an impedance increase during the scan. This peak is integrated over position to account for all the particles present in the sample, no matter how they are distributed. Then, the performance of the MNCs was evaluated by the sensor sensitivity (Σ), defined as for magnetoimpedance sensors. For a fixed frequency and type of particles, Σ is defined as the relative percentage increase in the impedance (*Z*) per unit mass of particles (m). For Z with and $Z_{0}$ without particles in the inductive sensor:

(1) $$\Sigma =\frac{1}{m}\frac{Z-Z_{0}}{Z_{0}}\times 100$$

The resolution of the sensor (R) is defined as the smallest change in mass to be resolved, where $\sigma_{\mathrm{base}}$ is the noise as the standard deviation of the signal over time when measuring a blank, and ΔZ is $Z-Z_{0}$:

(2) $$R=\frac{m\sigma_{\mathrm{base}}}{\Delta Z}$$

### 2.3. Bioconjugation of the Magnetic Clusters

To assess the performance of the MNCs in LFAs, they were firstly conjugated to neutravidin and used in an LFA strip with a test line of immobilized biotin. For this bonding, we used the carboxylic groups present in the MNC surface to link the neutravidin via the carbodiimide chemistry. For that purpose, 10 µL of the particles (previously sonicated for 5 min) was mixed with 100 µL of EDC (5 mg/mL, MES 50 mM, pH 6.00) and 100 µL of NHS (5 mg/mL, MES pH 6.00), both freshly prepared, and stirred for 10 min. Then, 100 µL of a neutravidin solution (1 mg/mL) was added and shaken for 4 h. To block the residual carboxyl groups on the particle surface, we added 100 µL of the blocking solution (1% BSA, PBS 10 mM, pH 7.4) for 30 min while agitating. The sample was then centrifuged at 14,600 rpm for 20 min, and 300 µL of the supernatant was removed. The pellet was resuspended in freshly prepared buffer (PBS 1 mM, pH 7.4).

**Figure 2:**
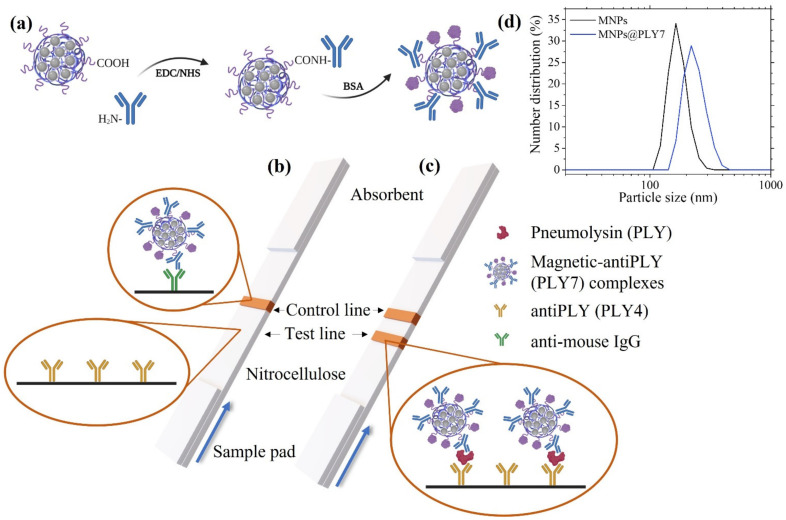
(**a**) Schematic illustration of the MNCs biofunctionalization with anti-pneumolysin antibody PLY7 via EDC/NHS chemistry. (**b**) Scheme of the lateral flow immunoassay, where specific antibodies against pneumolysin PLY4 (test line) and anti-mouse IgG antibodies (control line) are immobilized on the membrane. The blue arrow indicates the flow direction. In a blank sample, only the control line is visible. (**c**) Lateral flow assay for a sample containing PLY, which forms a complex with the MNCs-PLY7 and is captured at the test line by the PLY4 antibodies. (**d**) Hydrodynamic size distribution profile of the MNCs before (black) and after (blue) the bioconjugation with the PLY7 antibody.

In the immunoassay, a mouse monoclonal antibody (PLY7) against PLY was used as a selective detection antibody attached to the MNCs. Again, we used carbodiimide chemistry to activate the polyacrylic’s carboxylic groups at the MNC surface, as depicted in Figure 2a: 5 µL of the MNCs, previously sonicated for 5 min, was mixed with 100 μL of EDC (3.5 mg/mL in MES 50 mM, pH 6.00) and 100 μL of NHS (3.5 mg/mL in MES 50 mM, pH 6.00). The mixture was sonicated for 10 min. Then, 71 μL of the antibody (0.6 mg/mL) was added and left for 4 h in a refrigerated ultrasonic bath while mechanically agitated. After the conjugation, the residual activated carboxyl groups were blocked by adding 107 μL of the blocking solution (1% BSA in phosphate-buffered saline (PBS) 1 mM, pH 7.4) and sonicating for 1 h. Then, the mixture was centrifuged at 7000 rpm for 5 min, and 380 μL of the supernatant was discarded. The same amount of freshly prepared buffer (PBS 1 mM, pH 7.4) was used to resuspend the pellet.

The hydrodynamic size distribution and average ζ-potential before and after the conjugation process were obtained by dynamic light scattering (DLS) measurements performed in a Malvern Instruments Zetasizer Nano SZ (Malvern, UK) equipped with a solid-state He–Ne laser (wavelength 633 nm).

### 2.4. Preparation of the Immuno-Strips

For the LFA strip assembly, the nitrocellulose membrane was attached to an adhesive backing card. To create the test and control lines (see Figure 2b,c), the capture antibody (mouse monoclonal PLY4) and the anti-mouse IgG, respectively, both at a concentration of 1 mg/mL, were printed using a reagent dispenser. The latter consists of an automatic micropipette coupled with a micropositioner programmed to synchronize the micropipette dispensation with its motion. This printing method enables the deposition across the membrane at a rate of 0.1 µL/mm. Antibody binding to the nitrocellulose membrane is produced thanks to hydrogen bonding and hydrophobic and electrostatic interactions [42,43,44]. When the antibodies are dispensed, their initial attraction by the nitrocellulose involves the interaction between the carboxyl groups in the former and the nitro group dipoles in the latter. The final adsorption is further enhanced by the interaction among the nitrocellulose and hydrophobic domains within the protein. Thus, the antibodies are immobilized in the membrane strip.

After drying, the sample pad (which enables a controlled transfer of the sample to the membrane) and the absorbent pad (which acts as a wick and prevents backflow) were placed onto the backing card with an overlap of 2 mm with the membrane. Finally, single 5 mm wide strips were cut with a guillotine (MS Yosan 30).

Starting from a PLY standard stock solution (650 µg/mL), three solutions at concentrations of 12.1 µg/mL, 4.0 µg/mL, and 1.3 µg/mL were prepared by consecutive dilutions in PBS (1 mM, pH 7.4). To obtain the PLY concentrations tested in the LFAs (0 ng/mL for the blank sample; 13, 22, 30, 40, 60, 80, 120, 360, and 720 ng/mL for the rest), the adequate volume of these standard solutions was mixed with 20 µL of the MNCs-PLY7 conjugates and freshly prepared running buffer (RB) up to a final total volume of 100 µL. The RB contains 0.5% Tween20 in PBS (1 mM, pH 7.4).

The LFAs were carried out in dipstick format directly in the Eppendorf tube (2 mL). The sample pad is vertically introduced into the fluid that flows up the membrane, driven by capillary action. The performance of the LFAs relies on a sandwich format in which the detection antibody PLY7 and the MNCs form a complex with the target analyte if it is present. These complexes are trapped by the capture antibody at the test line, making it visible. The remaining sample will continue flowing all the way through the membrane, facing the control line, up to the absorbent (see Figure 2b,c for a detailed schematic). All the assays were performed in triplicate.

### 2.5. Magnetic Immuno-Concentration

Magnetic particles can enrich the sample by concentrating the labeled biomolecule into a smaller volume. It is especially interesting for applications with access to a sample volume larger than the 100 µL needed for an immunochromatographic assay. It is the case for urine samples, in which concentration can significantly aid in more sensitive detection when the amount of biomolecule in the fluid is meager.

To assess the capability of the particles to concentrate the biomolecule magnetically, we took a starting sample with 1.3 ng of PLY, diluted it, and then tried to recover the initial concentration by applying a magnetic gradient.

Initially, 1.3 ng of PLY was diluted into 80 µL of RB. From it, a collection of samples was obtained by successive addition of freshly prepared RB (ranging from x=0 µL to x=1500 µL). Then, 20 µL of the PLY7-MNCs conjugates was added, shaken, and left to sit for 15 min for the immunorecognition to take place. The x=0 sample is the reference one, with a concentration of 13 ng/mL. The rest have PLY concentrations ranging from 6.50 ng/mL to 0.81 ng/mL (see Table S1 for detailed information).

**Figure 3:**
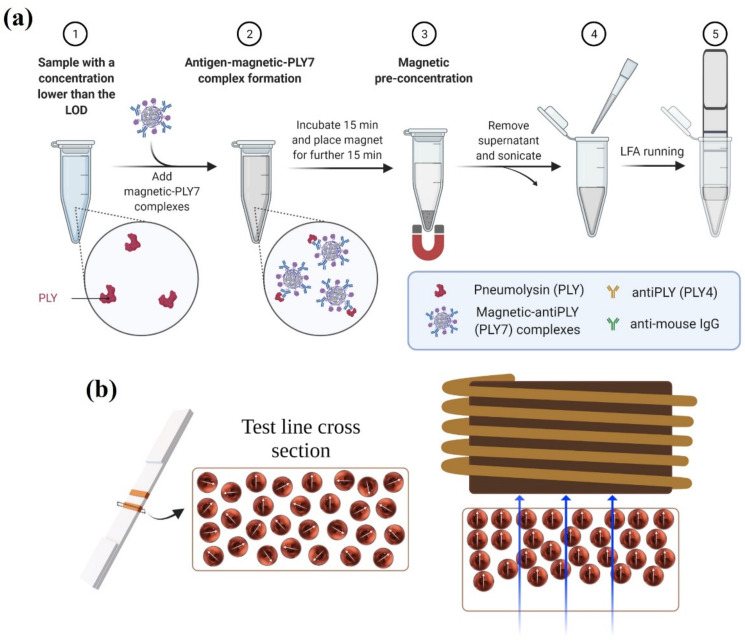
(**a**) Schematic illustration of the magnetic concentration procedure. (**b**) Schematic illustration of the MNCs distributed within the membrane before (left) and after (right) the relocation process. The blue arrows indicate the magnetic gradient direction.

The magnetic immuno-concentration procedure is depicted in Figure 3a. We used the most straightforward method for the enrichment. The Eppendorf tube was vertically placed on top of a NdFeB permanent magnet (N45, nickel-plated, S-70-35-N, Supermagnete); the magnetic field gradient was in the order of 64 mT/mm, as measured by a Hall probe (FW Bell, Model 4048, Orlando, FL, USA), for another 15 min to attract the PLY-labeled MNCs.

If all the conjugates are collected, the supernatant ideally should not contain any particles. In step 4 (see Figure 3a), the necessary supernatant was removed to leave a final volume of 100 µL. The magnetically retained pellet was resuspended and sonicated for 5 min. The sample concentration after this procedure was expected in all cases to be equal to 13 ng/mL. LFAs were carried out for these samples to check the method’s success.

### 2.6. Magnetic Relocation

Although magneto-inductive sensing is a form of non-contact, remote detection, the signal from the particles that are closer to the inductor is more intense. Hence, it is helpful to relocate the particles within the volume of the membrane towards the surface that faces the inductor. Three different LFA strips were subjected to magnetic relocation after being run as described in Section 2.4. A magnetic field gradient was applied perpendicular to the test line to demonstrate that the particles can be relocated within the membrane (see Figure 3b). For this purpose, 10 µL of RB was deposited on the test line and placed on one of the poles of an electromagnet. For 2 h, a direct current generated a magnetic field gradient of 0.32 mT/mm, as measured by a Hall probe (FW Bell, Model 4048). After the magnetic relocation, the signal of the LFAs was once again evaluated with the inductive sensor.

## 3. Results and Discussion

### 3.1. Characterization of the Magnetic Clusters

**Figure 4:**
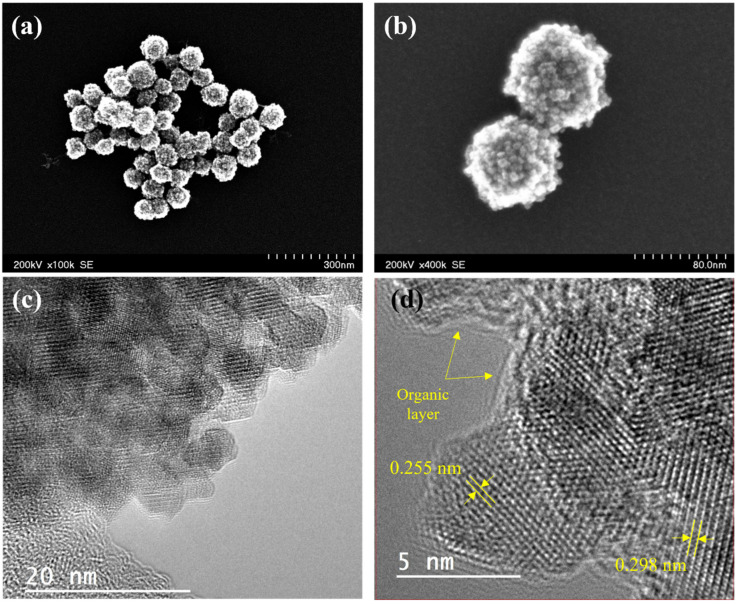
(**a**,**b**) STEM images of magnetic clusters. (**c**) HRTEM image of the magnetic nanoclusters with the organic layer at their surface. (**d**) HRTEM image with the interplanar distance corresponding to the family of crystalline planes (220) and (311).

Magnetite clusters were synthesized by a solvothermal method using a polyol process [39]. Sodium polyacrylate was used as a coating because the resulting clusters are relatively stable in suspension and display many carboxylate groups on the surface, which can be further functionalized. The size of the clusters was optimized to be below 100 nm using the ratio between the two solvents ethylene glycol and diethylene glycol. STEM images (see Figure 4a,b) confirm the formation of almost spherical magnetic clusters with a narrow size distribution with a mean size of 89 ± 2 nm and a standard deviation of 0.12 ± 0.03 nm.

HRTEM images in Figure 4c,d show the individual particles that make up the nanoclusters, with sizes that range from 4 nm to 7 nm. MNCs consist of a combination of individual agglomerated particles (attached by weak interactions of the polymeric coating) and aggregates (individual particles that share a surface). In Figure 4d, various particles appear trapped by an amorphous layer that corresponds to the organic polyacrylic acid on their surface. The interplanar crystal distances measured from the adjacent lattice fringes using a fast Fourier transform (FFT) are 0.298 nm, 0.255 nm, 0.220 nm, and 0.155 nm, which correspond to the planes with Miller indexes (220), (311), (400), and (440), respectively. These values agree with the standard card JCPDS Nº 19-0629 for magnetite.

**Figure 5:**
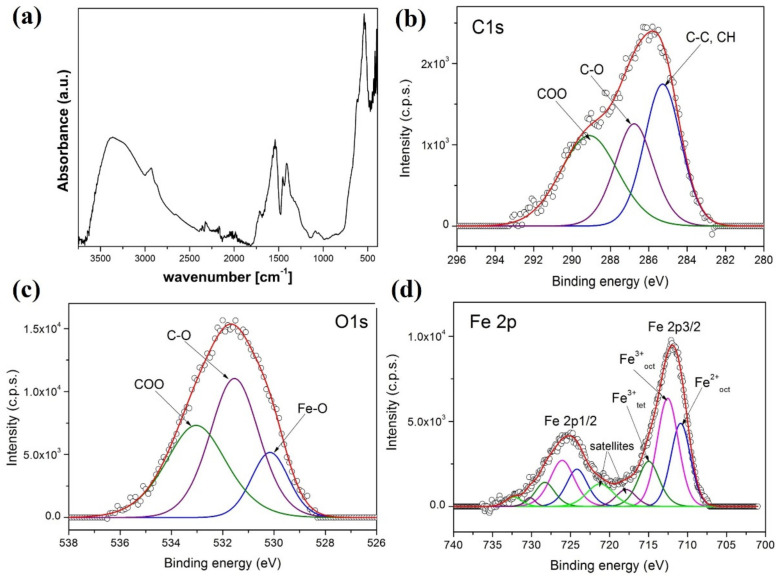
(**a**) FTIR spectrum of the magnetic nanoclusters coated with polyacrylic acid. (**b**) The high-resolution XPS spectra of C 1s, (**c**) O1 s, and (**d**) Fe 2p core-levels for the clusters coated with polyacrylic acid.

Figure 5a shows the Fourier transform infrared (FTIR) spectrum of the nanoclusters. A band at 540 cm^−1^ is due to Fe–O stretching, which can be ascribed to the spinel iron oxides. The peak at 1092 cm^−1^ stems from *v* (C–O), from ethylene glycol bound to the clusters. Bands at 1414 cm^−1^ and 1540 cm^−1^ are indicative of carboxylate groups (*v* (C=O)), both from polyacrylate as well as acetate bound to the surface. The bands at 2867 cm^−1^ and 2930 cm^−1^ are generic *v* (C–H) from the coating. Both surface-bound Fe–OH and carboxylic acid as well as hydroxyl groups (from ethylene glycol) give rise to the broad band between 3000 cm^−1^ and 3500 cm^−1^ (*v* (O–H)).

XPS analysis was used to demonstrate the successful preparation of magnetic clusters coated with polyacrylic acid. The high-resolution XPS spectra of C 1s, O 1s, and Fe 2p core-levels for the magnetic clusters are shown in Figure 5b–d, respectively. The spectra have been deconvoluted into components to obtain the best fit. The C 1s spectrum contains three components ascribed to C–C, CH groups (285 eV), COO (289.1 eV) from polyacrylic acid, and C–O (286.6 eV) from ethylene glycol, also present on the surface of the nanoclusters. The oxygen spectrum in Figure 5c exhibits three components assigned to the oxygen atoms from the carboxyl group COO of the polyacrylic acid (533 eV), from C–O (531.5 eV) from the ethylene glycol, and from Fe–O in the magnetite (530 eV). The Fe 2p spectrum is made up of the doublet Fe 2p-3/2 and Fe 2p-1/2. The best fit for the Fe 2p spectrum in Figure 5d contains the components corresponding to Fe^3+^ and Fe^2+^ ions and their corresponding satellites. From the Fe^3+^ and Fe^2+^ 2p peaks, an Fe^3+^/Fe^2+^ atomic ratio of 2 was obtained. This result confirms that the cluster contains magnetite nanoparticles. The surface chemical composition (atomic concentrations) of the magnetic clusters calculated from high-resolution XPS spectra has values of 26.6%, 48.5%, and 24.9% for C, O, and Fe, respectively. These results demonstrate the successful preparation of magnetite nanoclusters coated with polyacrylic acid.

**Figure 6:**
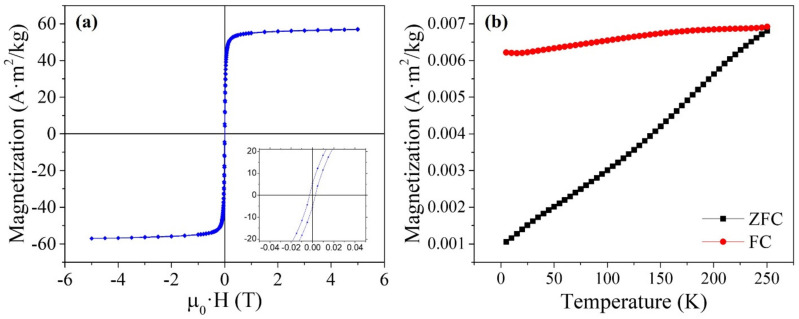
(**a**) Hysteresis loop of a powdered sample of the MNCs at 300 K. Inset: magnification of the curve at low fields. (**b**) ZFC-FC curves under a field of 2.5 Mt.

Field dependence of the magnetization of the MNCs sample at 300 K (Figure 6a) is not completely reversible, as shown in the inset (i.e., $H_{C}$ = 2.7 mT, $M_{r}/M_{S}$ = 0.09), suggesting the presence of constituent particles that are blocked at this temperature. This is confirmed by the ZFC-FC curves (see Figure 6b), where no irreversibility region is observed in all the explored temperature ranges [45]. This behavior can be ascribed to the presence of strong interparticle interactions confirmed by the flatness of the FC curve. Therefore, in each MNC, densely packed superparamagnetic and blocked particles coexist.

Regardless, the coercivity (width of the magnetization loop) and hysteresis loss (area of the loop) are small, and the initial susceptibility (slope of the magnetization curve near zero field) is high, with a value of 28 (dimensionless, SI units). The saturation magnetization ($M_{S}$) of the MNCs referenced to the magnetite mass was calculated by fitting the experimental data to the law of approach to saturation [46], yielding a value of 60 ± 2 A·m^2^/kg.

The MNCs were evaluated in the magnetic sensor, resulting in a sensitivity of 3.12% per mg of MNCs, as defined in Equation (1), which is better than our best result obtained with similar particles, 2.60% per mg of oleic acid-coated magnetic nanoclusters [47]. The resolution of the system, defined as in Equation (2), reached only 71 ng of MNCs, outperforming the 186 ng than can be distinguished with the oleic acid-coated magnetic nanoclusters.

### 3.2. Bioconjugation of the Magnetic Clusters

As a first attempt to assess the performance of the MNCs in LFAs, they were conjugated to neutravidin and used in an LFA strip with a test line of immobilized biotin. The optimal concentration of neutravidin for the bioconjugation to the MNCs was 1 mg/mL. The magneto-inductive signal obtained with the sensing device was 1.8 mΩ·mm, outperforming the best previous results obtained with lauric acid-coated magnetic nanoclusters: 1.1 mΩ·mm for 2 mg/mL of neutravidin and 0.65 mΩ·mm for 1 mg/mL [47]. These results advised the use of the present MNCs for PLY detection.

DLS measurements confirmed the successful conjugation of the MNCs to PLY7 (see Figure 2d). This technique allows the comparison between the nanoparticle hydrodynamic sizes before and after the reaction. The hydrodynamic diameter of the MNCs before the conjugation was 210 nm (PDI 0.3). A comparison of this result with the value obtained by STEM, which gave a mean cluster size of 89 nm, indicates some agglomeration among the clusters, forming chains or agglomerates of 2–3 MNCs, as seen in Figure 4b. DLS sizes are usually about 15% larger than TEM sizes due to the surfactant and hydration layers. Agglomeration can positively impact the detection because it increases the magnetic moment per biomolecule [48]. After the PLY/antibody conjugation, the new hydrodynamic size increased up to 237 nm (PDI 0.1). Taking into consideration the typical size of 10 nm for IgG type antibodies [49], this increase indicates the success of such attachment.

ζ-potential was used to assess the stability of the nanoclusters and the MNCs-PLY7 complexes. The values obtained were −35 mV and −29 mV for the bare clusters and the MNCs-PLY7 complexes, respectively. These values are typical of sufficiently strong electrostatic repulsions to provide colloidal stability of the particle solution [50].

### 3.3. Lateral Flow Strips Reading-Out

**Figure 7:**
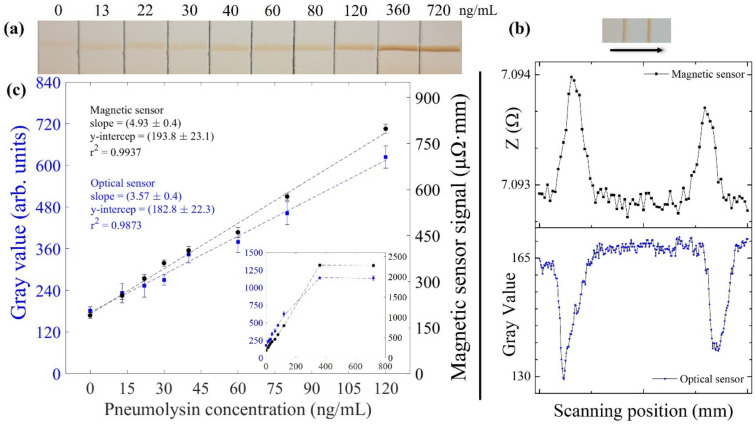
(**a**) Image of the test lines of some LFAs covering the calibration range of PLY. The PLY concentration, shown above each strip, increases from left to right, as does the intensity of the test line. (**b**) LFA with 120 ng/mL of PLY and its magnetic (black) and optical (blue) signals obtained when scanning in the direction of the arrow. The left peak corresponds to the test line and the right one to the control line. (**c**) Calibration curves for the optical (blue) and inductive signals obtained in the linear range of PLY concentration. Inset: Calibration curves for all PLY concentrations tested. Both graphs show the mean value, and the error bars represent their standard deviation. Axis and units are the same for graph and inset.

Once the tests were run and dried overnight, their optical and magnetic signals were measured and analyzed. Figure 7a shows the test lines of all the LFAs for one of the three series, where the PLY concentration increases from left to right. As can be seen, the test line intensity increases in this direction, in the usual way for a sandwich format immunoassay. Figure 7b shows the LFA with 120 ng/mL and its magnetic and optical signals. The two peaks in the records correspond to the MNCs present at the test line (left) and control line (right). The optical (blue) and magnetic (black) calibration curves comprising the whole range of concentrations tested are presented in the inset of Figure 7c. Both signals increase with the PLY concentration up to saturation, which is reached when no more immobilized PLY4 antibody is available for binding the PLY-PLY7-MNC conjugates at the test line. Figure 7c shows the linear range of these calibration curves with the fitting parameters of the experimental data. The magnetic signal shows a better correlation factor. Following the Eurachem Guidelines [51], the limit of detection (LOD) and the limit of quantification (LOQ) were calculated with the $\sigma_{\mathrm{blank}}$, the standard deviation of the concentration obtained for more than 10 independent measurements of the blank, as

(3) $$\mathrm{LOD}=3.3\times \sigma_{\mathrm{blank}}\;\mathrm{LOQ}=10\times \sigma_{\mathrm{blank}}$$

The former is the lowest analyte concentration that can be reliably distinguished from the blank, whereas the latter is the lowest concentration at which the quantifying method performance is acceptable. Hence, an LOD of 6.3 ng/mL for the magnetic method and 10.8 ng/mL for the optical method are found. The LOQ were 19.0 ng/mL and 32.7 ng/mL for the inductive and optical signals, respectively. Both values and the superior correlation confirm the better performance of the inductive sensing platform.

### 3.4. Magnetic Immuno-Concentration

**Figure 8:**
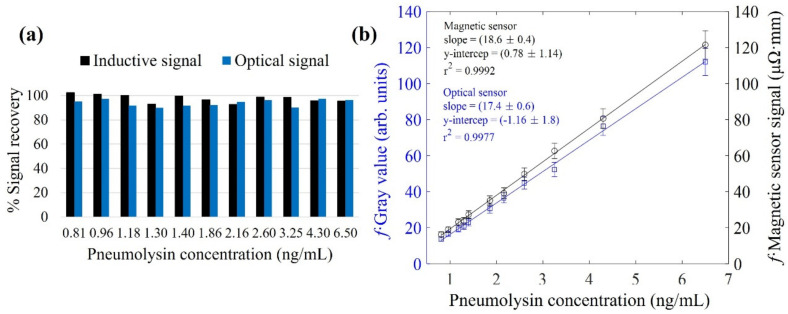
(**a**) Signal recovery percentage for the optical (blue) and inductive (black) sensor. (**b**) Calibration curves obtained for the optical and the inductive sensors with the concentrated values. The error bars are calculated as the sum of the standard deviations of the linear regression of the calibration curve together with the error in the pipetting of each dilution volume.

To evaluate the magnetic concentration process, we took the following steps. From a known concentration solution, we obtained a set of samples diluted with different volumes and magnetically concentrated up to the same initial concentration (see Table S1 in Supplementary Materials). We then defined the magnetic concentration efficiency by the signal recovery percentage. This value refers to the ratio between the signals of the concentrated sample and the undiluted one. Both values might be the same if the concentration is successfully carried out. Otherwise, if some magnetic material is removed with the supernatant, the MNCs retained at the test line will decrease, as will their signal. Figure 8a shows these signal recovery percentages for all the concentrated samples tested. The percentages are (98 ± 3)% and (94 ± 3)% for the inductive and optical methods, respectively. Thus, these figures indicate that the magnetic concentration procedure is validated.

To evaluate the new figures of merit of the technique due to magnetic concentration, we represent on the vertical axis of Figure 8b the obtained signal multiplied by a concentration factor f that considers the relationship between the intermediate and final volumes of the tested samples. This factor is defined as $f=V_{f}/V_{i}$, where $V_{f}$ and $V_{i}$ are the final (100 μL) and the intermediate volumes, respectively (see Table S1 in Supplementary Materials). This factor allows us to relate the signal obtained with the magnetic and optical methods to the value of the intermediate concentration. The calculated calibration curves with their fitting parameters are shown in in Figure 8b. The LODs have 0.2 ng/mL and 0.6 ng/mL values for the inductive and optical systems, respectively. The new LOQs decreased to 0.7 ng/mL for the inductive sensor and 1.7 ng/mL for the optical one. This means that by taking advantage of the magnetic concentration, it is possible to clearly improve the LOD and LOQ of the system with both methodologies. Further improvement can be expected if the drag force is optimized by a specifically designed array of permanent magnets, as proved in [52]. These new figures of merit are achieved thanks to the magnetic character of the labels used.

Remarkably, the optical evaluation of the LFAs performed with just a mobile phone show excellent LOD and LOQ thanks to the magnetic concentration. These results show promise for diagnostic testing in harsh locations or locations with few resources. The inductive sensor gives more precise and reliable quantitative results. The staining or aging of the supporting nitrocellulose membrane does not interfere with the magnetic signal, nor does the ambient light. Consequently, the reproducibility of the inductive measurement technique allows storage and reuse of the calibration data for further sample evaluation.

### 3.5. Magnetic Relocation

Magnetic relocation was explored to further increase the sensitivity of the technique. Once the LFAs were run, a magnetic field gradient was applied at the test line to relocate the MNCs closer to the sensing region for LFAs, with 6.5 ng/mL, 40 ng/mL, and 60 ng/mL of PLY resulting in an increase in the signals of 18%, 20%, and 28%, respectively.

This technique deserves further investigation. There are different bonds present with different binding energies, which could determine which one breaks: the bond between the capture antibody and the membrane, between the capture antibody and the antigen, and between the particles and the detection antibody. Although the former seems most likely, its confirmation requires further studies. The characteristics of the nanoparticles are key. For example, high magnetization and susceptibility are determining factors in their response to the applied relocation field gradient. It will also be worth studying the efficiency of the relocation in terms of the intensity of the magnetic gradient and time. Nevertheless, these preliminary results show the potential of magnetic relocation to further improve the LOD within an integrated device.

## 4. Conclusions

Pneumolysin quantification was developed via a combination of magnetic labeling in a lateral flow immunoassay and an inductive sensor. Spherical iron oxide nanoclusters with mean sizes of 89 nm were synthesized by a polyol method. Their capping with polyacrylic acid enabled their biofunctionalization for the specific recognition of pneumolysin. The magnetic immunoassay was then calibrated with standard PLY solutions. The read-out of the test lines was dual: magnetic (by inductive sensing) and optical (by image analysis with a phone camera). The inductive reader yielded a larger linear range and better correlation factor, limit of detection, and limit of quantification. Still, the figures of merit of the mobile phone method were remarkable, taking into account the availability of such a device. More interestingly, the magnetic character of the clusters allowed us to considerably improve these figures of merit: the magnetic concentration procedure set new LODs at 0.2 ng/mL and 0.6 ng/mL for the inductive and optical sensors, respectively. Additionally, the magnetic relocation of the particles within the membrane further increased the signal of the LFAs by 20%.

The system has potential for pneumococcal pneumonia diagnosis in harsh environments using just a mobile phone camera. The magneto-inductive sensor allows further increase in the sensitivity of the method. In any case, both approaches overcome the current drawbacks in etiological diagnosis, such as false-negative cultures, detrimental and inconclusive X-rays, especially in children, and the indiscriminate use of antibiotics.

## Data Availability

Not applicable.

## Supplementary Materials

The following supporting information can be downloaded at: https://www.mdpi.com/article/10.3390/nano12122044/s1; Table S1: Samples tested in the magnetic concentration process with their initial PLY, the volume of RB added to obtain the diluted sample, and their diluted concentration.
