# Supplementary material for: Magnetic Nanoclusters Increase the Sensitivity of Lateral Flow Immunoassays for Protein Detection: Application to Pneumolysin as a Biomarker for Streptococcus pneumoniae
Source: Nanomaterials (Basel). 2022 Jun 14;12(12):2044. doi: 10.3390/nano12122044 (PMC9228753; doi:10.3390/nano12122044)
Supplement: Supplementary file 1 [file nanomaterials-12-02044-s001.zip › nanomaterials-1742197-supplementary.pdf]

nanomaterials

MDPI

Supplementary Materials

Magnetic Nanoclusters Increase the Sensitivity of Lateral Flow Immunoassays for Protein Detection: Application to Pneumolysin as a Biomarker for *Streptococcus pneumoniae*

María Salvador [1,2], José Luis Marqués-Fernández [1], Alexander Bunge [3], José Carlos Martínez-García [1], Rodica Turcu [3], Davide Peddis [2,4], María del Mar García-Suárez [5], María Dolores Cima-Cabal [5] and Montserrat Rivas [1,*]

[1] Department of Physics & IUTA, University of Oviedo, Campus de Viesques, 33203 Gijón, Spain; salvadormaria@uniovi.es (M.S.); uo254204@uniovi.es (J.L.M.-F.); jcmg@uniovi.es (J.C.M.-G.)
[2] Institute of Structure of Matter—National Research Council (CNR), 00016 Monterotondo Scalo (RM), Rome, Italy; davide.peddis@unige.it
[3] National Institute for Research and Development of Isotopic and Molecular Technologies, 400293 Cluj-Napoca, Romania; alexander.bunge@itim-cj.ro (A.B.); rodica.turcu@itim-cj.ro (R.T.)
[4] Department of Chemistry and Industrial Chemistry, Università degli Studi di Genova, 16146 Genova, Italy
[5] Escuela Superior de Ingeniería y Tecnología (ESIT), Universidad Internacional de la Rioja, UNIR. Avenida de la Paz, 137, 26006 Logroño, Spain; mar.garcia.suarez@unir.net (M.d.M.G.-S.); dolores.cima@unir.net (M.D.C.-C.)
* Correspondence: rivas@uniovi.es

Table S1. Samples tested in the magnetic concentration process with their initial PLY, the volume of RB added to obtain the diluted sample and their diluted concentration.

Sample	Initial PLY (ng)	Volume Added (µL)	Volume of the Diluted Sample (µL)	Concentration of the Diluted Sample (ng/mL)
Reference	1.3	0	100	13.00
1	1.3	100	200	6.50
2	1.3	200	300	4.33
3	1.3	300	400	3.25
4	1.3	400	500	2.60
5	1.3	500	600	2.17
6	1.3	600	700	1.86
7	1.3	800	900	1.44
8	1.3	900	1000	1.30
9	1.3	1000	1100	1.19
10	1.3	1250	1350	0.96
11	1.3	1500	1650	0.81
